# Knowledge of autism among students at a South African Institute of Higher Education

**DOI:** 10.4102/hsag.v29i0.2301

**Published:** 2024-05-31

**Authors:** Marguerite De Jongh, Heidi A.M. Mapisa

**Affiliations:** 1 Department of Speech-Language Pathology and Audiology, School of Health Care Sciences, Sefako Makgatho Health Sciences University, Ga-Rankuwa, South Africa

**Keywords:** autism spectrum disorder, curriculum, identification, assessment, intervention, speech-language therapists, speech-language pathology, audiology

## Abstract

**Background:**

Autism is a significant concern because of the increase in the prevalence of the disorder. University healthcare students might not all be adequately prepared to serve autistic individuals. Hence, there is a need in the South African context for information on healthcare practitioners’ knowledge of general aspects, diagnosis and management of autism.

**Aim:**

To determine current knowledge on autism among speech-language pathology and audiology (SLP & A) students at a South African Higher Education Institution.

**Setting:**

The study was conducted among 65 second, third and fourth year students at the SLP & A Department of a South African Higher Education Institution.

**Methods:**

A descriptive quantitative design utilising an online questionnaire was used to gather the quantitative and, to a lesser extent, qualitative data. Descriptive measures were used to analyse and summarise the data.

**Results:**

Participants mainly understood autism’s fundamental symptoms and comorbidities, early intervention, team management and speech-language therapist (SLT) duties. Students were found to have little awareness of autism’s prevalence, causes, diagnosing experts, intervention methods and treatment. Participants felt uncomfortable treating autistic people owing to a lack of clinical exposure. Participants want further training.

**Conclusion:**

Students reported the need for additional training on autism, including its identification, diagnosis, assessment and treatment. It is recommended that the study be replicated at other institutions to impact other curricula.

**Contribution:**

This research article provides input for enhancing the curriculum for Health Science Departments in Higher Education Institutions.

## Introduction

Autism is a ‘spectrum’ disorder and refers to a group of neurodevelopmental conditions characterised by a disparity in the type and severity of persistent symptoms (Chansa-Kabali, Nyoni & Mwanza 2019). These symptoms include insufficient social interaction skills, lack of communication, as well as restricted and repetitive behaviour (Chansa-Kabali et al. 2019).

Autism was previously regarded as a disorder that mostly occurred in well-resourced countries that are technologically advanced (Bakare et al. 2015). One in every 54 individuals globally presents with autism (Kuo 2022). However, in sub-Saharan African countries, population-based prevalence studies are absent (Franz et al. 2018). Using the United States’ prevalence ratio as a point of reference, an estimated 270 000 people in South Africa might have autism. This might indicate a rise in autism in the last two decades in low- and lower-middle-income countries (Ruparelia et al. 2016).

Ruparelia et al. (2016) stated that research from sub-Saharan Africa, including South Africa, is limited. Scanty studies indicate low awareness and a lack of general knowledge on autism and associated conditions. Insufficient understanding of the incidence and prevalence among healthcare practitioners in sub-Saharan African countries, including the South African context among healthcare practitioners, was also noticed. This might impact the diagnosis and intervention of individuals with autism and has far-reaching consequences for service delivery (Franz et al. 2018; May et al. 2017).

According to McCarthy et al. (2020) and Mazurek et al. (2017), healthcare providers, such as speech-language pathologists and audiologists (SLP & As), are perfectly positioned to meet the needs of individuals with autism, but they often lack understanding because of limited training. McCarthy et al. (2020) likewise reported that 90% of school-based speech-language therapists (SLTs) support autistic children as part of their caseload, but they might not have been equally prepared as they received varied input and training on managing autistic individuals at a tertiary level. These varying educational backgrounds of students may result in school-based SLTs using programmes and approaches that may have little or no supporting evidence on the effectiveness of these programmes. According to McCarthy et al. (2019), SLTs believed that the coursework they received during their undergraduate programmes did not prepare them to manage these individuals as they did not receive enough exposure to autism before graduating. Consequently, they lack foundational knowledge of the disorder, impacting their preparedness and ability to provide evidence-based practice to autistic children. This study further emphasised the need to include autism as a required component of undergraduate and postgraduate programmes to meet preparation expectations. However, researchers such as Franz et al. (2018) indicated a gap in the research on how healthcare practitioners realise and manage autism. The scoping review by Franz et al. (2018) indicated that no research was published on SLTs’ knowledge of autism in the South African context. Van Biljon, Kritzinger and Geertsema (2015) also stated this. Likewise, in the SLP & A Department at a certain institution of higher education, no studies have previously been conducted on students’ knowledge of autism. Hence, students in this department may have had limited exposure and time to engage with content related to autism as students currently follow a dual programme and autism content is presented as a unit embedded within a module. All of the aforesaid may impact their understanding of autism. Research from Franz et al. (2018), May et al. (2017) and McCarthy et al. (2020) additionally highlighted healthcare providers’ lack of knowledge related to the causes of autism, the comorbidities associated with autism as well as specific assessment and intervention that needs to be implemented for individuals with autism. Therefore, it is significant that the researchers determine what is known about autism (Franz et al. 2018), namely the possible causes, that is genetic and environmental factors, genetic mutations and disorders, metabolic imbalances, and a history of infections or exposure to medications (Cherney & Seladi-Schulman 2018; Lubin 2015).

In addition, comorbidity between autism and other mental health disorders, such as attention deficit hyperactivity disorder (ADHD) (May et al. 2017), has implications for assessment, diagnosis, intervention and quality of life (Ellias & Shah 2019; Lord & Bishop 2015). The Diagnostic and Statistical Manual of Mental Disorders, Fifth Edition (DSM-5) classifies the features of autism and assists clinicians in describing how autism affects individuals’ communication and social behaviour.

Utilising the DSM-5 criteria in assessment assists the SLT in identifying the symptoms and determining the severity of the communication disorder, consequently affecting the management of individuals with autism (Lord & Bishop 2015). Assessment of autism should be comprehensive (Goldstein & Ozonoff 2018), and the diagnosis must be made by trained medical professionals, including physicians and psychiatrists. However, healthcare professionals (HCPs), such as nurses, occupational therapists (OTs), physical therapists (PTs), SLTs, psychologists as well as parents and special educators play a role in the early identification of autism as this will positively impact the management of autism (Bultas & Koetting 2014).

The World Health Organization (WHO) (2019) states that providing intervention during early childhood is vital to promote optimal development and well-being of children with autism and that a multidisciplinary team should be involved in managing autism because the individual with autism may present with difficulties in different areas (Oommen et al. 2017). The treatment of autism may include different approaches, such as nonpharmacological and cognitive behavioural approaches, parental training, applied behavioural analysis and pharmacological intervention (WHO 2019). However, Hayat et al. (2019) reported an overall lack of knowledge among healthcare professionals on utilising these treatment approaches.

The role of SLTs as part of the multidisciplinary team is to identify, assess and treat communication disorders in individuals with autism who display difficulties with speaking, listening and understanding language and social skills (American Speech Language Association [ASHA] 2017). The WHO (2019) additionally indicates that individuals with autism and their caregivers should be assisted and provided with relevant information on applicable services, referrals, treatment options and support. This assistance will help caregivers cope with the condition and manage concerns related to their children’s behaviour (Shulman et al. 2020).

Freeman Barnett (2022), however, identified a gap regarding SLTs’ preparedness in training and managing individuals with autism. This study, therefore, aimed to determine knowledge of autism among SLP & A students at a South African Higher Institution of Education as this will establish specific information and training to be presented to SLPA students to improve their knowledge, skills and competencies.

## Research methods and design

### Study design

A quantitative descriptive design was used by gathering online data through a self-administered questionnaire. This design provided insight into the knowledge of autism in SLP & A students. The study was mainly quantitative, but a few open-ended questions were included to clarify some of the information provided by the participants. A questionnaire was compiled by adapting the ‘Knowledge Assessment on Autism Spectrum Disorder Questionnaire’ by Ilg, Hauth-Charlier and Clement (2012). Furthermore, other relevant literature from studies of Lord and Bishop (2015), Lubin (2015), Ellias and Shah (2019), Bultas and Koetting (2014), and the WHO (2019) were used to include additional questions related to assessment, diagnosis and treatment.

### Setting

The study was conducted at an SLP & A Department at a South African Higher Education Institution.

### Study population and sampling strategy

Convenience sampling was utilised because the participants were readily available and willing to participate. The study focused on second-, third- and fourth-year students. These year groups were chosen as autism lectures were only introduced from the second year onwards in the specific department. Initially, 94 second- to fourth-year students registered for BSLPA were identified.

All year groups (second to fourth year) were invited separately to informal sessions to explain the potential research. Blind carbon copy (Bcc) lists were used to send emails to multiple recipients to avoid using the ‘reply all’ function and ensure anonymity. Aspects related to the research on how to access the Google Forms link and complete the questionnaire as well as the study aims, rationale and procedures were explained to the students of different year groups during separate Zoom information sessions before the research commenced. A link (https://bit.ly/3NdOQ9a) was created and emailed to all potential second- to fourth-year participating students. The link directed the participants through the Google Drive website, enabling students to access the questionnaire.

### Data collection

Shortly after the information sessions, the study commenced, and students completed the online self-reporting questionnaire using the Google Forms link. The questionnaire was distributed through Web-intercept, making it easier for participants to access and complete the questionnaire. It took approximately 10 min – 15 min to complete the questionnaire.

Validity and reliability were assured on different levels. The questions in the data collection tool corresponded with the guidelines presented by Taherdoost (2016) regarding questionnaire adaptation, validation and reliability. The knowledge assessment questionnaire on autism spectrum disorder by Ilg et al. (2012) was adapted for this study to coincide with the construct being tested: students’ knowledge of autism. The questionnaire only included the most pertinent questions about autism to avoid confusion and permit content validity. For face validity, a senior researcher provided feedback on the content and clarity of the questionnaires. Reliability was established by comparing item scores. If a questionnaire was completed in full, it was used for data analysis purposes.

Bias was minimised by ensuring the instrument was culturally sensitive and measured what it was supposed to measure (Taherdoost 2016). This was done by warranting that the questions in the questionnaire were not related to specific cultures and that the same procedures for collecting data were implemented throughout.

### Data analysis

Completed questionnaires were sent back directly to the researchers’ email accounts. Once the participants submitted their responses, Google Forms automatically created a summary of all the responses. Descriptive measures were used to analyse and summarise the quantitative data. The responses were also entered on a Microsoft Excel spreadsheet. The qualitative data (responses to open-ended questions) were initially quantified. All similar responses were categorised, quantified and presented in tables. Responses were arranged from the highest to the lowest percentages. The categories were compared to find patterns and similarities between the responses and to identify possible theme(s) by using inductive analysis. The qualitative component assisted researchers in gaining insight and effectively linking the qualitative data with the quantitative data.

### Ethical considerations

The research commenced once permission was obtained from the Sefako Makgatho Health Sciences University Research Ethics Committee (SMUREC/H/102/2020: UG). A consent statement was included in the online questionnaire. By completing the questionnaire, the participants consented to participate in the study. Therefore, all second- to fourth-year students registered for SLP & A at the specific Health Sciences University, who completed the consent statement and questionnaire, were included in the main study. Students not interested in participating exited Google Forms. Participants were informed of the purpose of the research, that they could withdraw at any given time and that their anonymity was ensured. Information remained confidential and anonymous as Google Forms did not require participants to provide personal or identifiable information. The researchers followed ethical guidelines where participants could decide at any given time to terminate the completion of the questionnaire. Students were also informed that the study was in the participants’ best interests as the results could warrant curriculum changes in the specific SLP & A Department that could increase students’ future knowledge and management of autism. The researchers followed ethical guidelines by treating all participants equally, fairly, and with respect and dignity.

## Results

A pilot study was conducted before the main study commenced. The results indicated that the content of the questionnaire was adequate. Thereafter, the main study commenced with 65 participants.

The demographic results of the *main study* are presented in Table 1.

**TABLE 1:** Distribution of demographic information.

Characteristics	Description	Number of responses provided	Percentage of respondents
Year of study	Second year	20	-
	Third year	20	-
	Fourth year	24	-
Participants repeating a year(s)	Yes	16	25
	No	48	75
Level (year) of study repeated	Second year	9	60
	Third year	6	40
	Fourth year	0	0
Gender	Female	52	80
	Male	13	20

Most participants were fourth-year students (*n* = 24), followed by the second- and third-year groups with 20 participants (*n* = 20). Twenty-five percent of participants indicated that they repeated either their second or third year. Most participants were females (80%), and the participants’ ages ranged from 19 to 28 years, with the average age being between 20 and 24 years.

The results further indicated that of the 93 students (second- to fourth-year students) who received the survey, 65 students responded. Hence, the return rate was 69.9%, which is considered a good response rate. This might indicate that the students were interested in the topic or had a personal reason for completing the questionnaire. However, some questions were left unanswered; thus, the sample size for questions varied, as indicated in Table 2.

**TABLE 2:** Knowledge of the general and diagnostic features of autism.

Questions and statements	Number of responses per questions and statement	Yes		No		Unsure	
		*n*	%	*n*	%	*n*	%
**Objective 1: General knowledge of diagnostic features of autism**							
Have you ever watched a TV documentary or read articles on autism?	65	55	85	10	15	-	-
Do you have a family member or know or have seen someone with autism before?	65	27	42	38	58	-	-
Autism only exists in childhood.	65	10	15.4	45	69.2	10	15.4
Autism is a developmental disorder.	65	50	77	11	17	4	6
Autism is a lifelong condition.	65	53	81.5	1	1.5	11	17
Autism is related to spiritual problems.	65	4	6	53	82	8	12
Is autism a genetic disorder?	65	43	66.2	11	16.9	11	16.9
People with autism have attention problems.	65	56	86	5	8	4	6
People with autism have difficulties with language production and comprehension	65	55	84.6	7	10.8	3	4.6
Do people with autism have communication difficulties?	65	60	92	3	5	2	3
People with autism have social interaction difficulties.	65	61	94	2	3	2	3
Do people with autism have any intellectual disabilities?	64	41	64	13	20	10	16
People with autism have associated behavioural and emotional difficulties.	65	58	89	2	3	5	8
Do the symptoms of autism vary from one person to another?	65	59	91	4	6	2	3
Is autism more common in females than in males?	64	7	11	29	45	28	44
The cause of autism is mostly unknown.	64	27	42.2	14	21.9	23	35.9
Can autism result from defective early brain development?	64	38	59.4	4	6.2	22	34.4
Can autism result from exposure to heavy metals and environmental toxins?	65	17	26	19	29	29	45
**Objective 2: Knowledge of assessment**							
Can autism be diagnosed at any developmental stage?	64	41	64	11	17	12	19
Children with autism can only be diagnosed at or after 3 years.	65	29	45	15	23	21	32
Can speech-language therapists make an autism diagnosis?	65	29	44.6	29	44.6	7	10.8
Behaviour and general development of a child are important to consider when making an autism diagnosis.	65	58	89	2	3	5	8

*Source:* Ilg, J., Hauth-Charlier, S. & Clement, C., 2012, *Knowledge assessment questionnaire on Autism Spectrum Disorders*, Research Gate, University de Strasbourg, Strasbourg

TV, television.

The results concerning speech-language pathology students’ knowledge of autism are discussed according to the study’s objectives.

The results for the first objective reflect on SLP & A students’ general knowledge of the diagnostic features of autism. In contrast, the second objective signifies their knowledge of the assessment of autism. A summary of the results is presented in Table 2.

The results for Objective 1 and Objective 2 represent the participants’ general knowledge of the diagnostic features of autism, including the causes, prevalence, symptoms, signs and comorbidities. Eighty-five percent (*n* = 55) reported having heard of autism from watching television documentaries and reading about the disorder, and 42% (*n* = 27) indicated that they either have a family member with autism or know of someone with autism. Participants who reported having some knowledge about autism learned about it through self-directed learning utilising the university library and the internet. Seventy-seven percent (*n* = 50) of the participants correctly indicated that autism is a developmental disorder and not only exists in children, whereas 15% (*n* = 10) believed that autism only existed in children, and another 15% were ‘unsure’. Zerbo et al. (2015) likewise indicated that healthcare professionals in their study believed that autism only affected children. Concerning gender prevalence, 45% (*n* = 29) indicated that autism was more common in males, and 44% (*n* = 28) participants were ‘unsure’. According to Anagnostou et al. (2014), males with autism outnumber females by as much as 4:1. These responses indicated limited knowledge by students.

The results also indicated that 42.2% (*n* = 27) agreed that the cause of autism is unknown, and 58% of participants (*n* = 38) either did not agree or were uncertain. Mohamed et al. (2015) found that both hereditary factors and exposure to different environmental factors play some role in the development of autism. Sixty-six percent (*n* = 42) of participants agreed that genetics is one of the risk factors associated with the development of autism. The results on the causes of autism revealed limited knowledge. However, 89% (*n* = 58) of the participants agreed on the different symptoms associated with autism, such as communication and social interaction difficulties, as well as behavioural and emotional problems.

The findings concerning Objective 2, that is assessment, indicate that only 45% (*n* = 29) of the 65 participants correctly reported that autism could only be diagnosed at or after 3 years of age. The remainder of the participants (55%) either did not agree or were ‘unsure’. The responses to the first question (*Can autism be diagnosed at any developmental stage?*) and the corresponding statement (*Children with autism can only be diagnosed at or after 3 years.*) do not correlate even though the same feature is being assessed, that is the age of autism diagnosis. Hence, the responses indicated that participants were unsure about the age of diagnosis in individuals. Eisenhower et al. (2020) indicate that children with autism can be diagnosed by 36 months of age, but the median age of diagnosis may only be at 52 months.

Participants were also required to indicate whether SLTs would be responsible for diagnosing autism. Forty-four percent (*n* = 29) correctly said ‘no’, and the remainder (*n* = 36) either said ‘yes’ or were ‘unsure’. This exposes a knowledge gap. According to Anagnostou et al. (2014), only physicians can determine if a child meets the autism criteria and make the diagnosis by applying the DSM-5 criteria (American Psychiatric Association [APA] 2013). Furthermore, most participants had some knowledge (*n* = 58) when asked if behaviour and general development should be considered when diagnosing autism.

The participants also had to select the healthcare professional(s) making the final diagnosis. Twenty-three percent (*n* = 15) incorrectly believed that autism is diagnosed by SLTs (23%). Speech-language therapists would identify and provide diagnostic input to physicians (Ellias & Shah 2019), but only trained medical or mental health professionals or clinicians such as developmental paediatricians, child neurologists, child psychologists and psychiatrists can diagnose autism (Bultas & Koetting 2014).

The quantitative results concerning SLPs’ knowledge of the management of autism (Objective 3) were obtained using both a Likert-type scale and closed-ended questions. A few open-ended questions were used for the qualitative data. The quantitative and qualitative results are presented in Table 3, Table 4 and Table 5.

**TABLE 3:** Treatment of autism.

Questions and statements	Number of responses per question and statement	Yes		No		Unsure	
		*n*	%	*n*	%	*n*	%
Is there any cure for autism?	64	3	5	50	78	11	17
Are there any alternative ways to manage autism?	64	60	93	3	5	1	2
Traditional healers can treat autism.	64	2	3	42	65	20	31
Behavioural therapy is one of the treatment options of autism.	64	58	91	2	3	4	6
Can early intervention help minimise the symptoms of autism?	64	57	89	3	5	4	6
Parent and/or family involvement is important in the management of autism.	64	63	98	0	-	1	2
Speech-language therapists are part of the autism management team.	64	62	96	1	2	1	2

**TABLE 4:** Response categories: Speech-language pathology and audiology students’ knowledge of the management of autism.

Response categories: Knowledge of autism management	*N*	%
**A: Response categories: Why autism requires a team approach**	**41**	
Management of all functional areas	31	75.6
Collaboration	4	9.8
Implementation of a holistic approach	4	9.8
Complexity of the disorder	2	4.8
**B: Response categories: Roles of SLTs in the management of autism**	**38**	
Improve language and communication difficulties	31	81.0
Improve social interaction	6	16.0
Appropriate referral and intervention planning	1	3.0
**C: Response categories: Participants’ feelings associated with the management of individuals with autism**	**24**	
Limited experience and training on autism management	16	66.7
Personal interest and enough experience on autism management	8	33.3

SLT, speech-language therapist.

**TABLE 5:** The importance of receiving additional training on autism.

Response categories: Reasons why it is important to receive additional training	*N*	%
Improved competence in assessing and managing individuals with autism	23	62.2
Enhanced (holistic) knowledge on the disorder	14	37.8

**Total**	**37**	**100.0**

The quantitative data on participants’ knowledge aimed at the different treatment options available for autism, regarding whether autism was curable, the effectiveness of early intervention, and the team members that should be involved in treating individuals with autism, are presented in Table 3.

Participants were asked to indicate whether there is a cure for autism. Most participants (*n* = 50) correctly specified ‘no’, and the remainder (*n* = 15) either said ‘yes’ or were ‘unsure’, indicating adequate knowledge that there was no cure for autism. Furthermore, 91% (*n* = 58) of the participants correctly chose ‘yes’, indicating that behavioural therapy is one of the treatment options available for autism, and also acknowledged that rigorous behavioural interventions undertaken early in life are considered the current gold-standard treatment for behavioural challenges as reiterated by Masi et al. (2017).

Most of the participants (*n* = 57) also chose ‘yes’, indicating that early intervention could minimise the symptoms of autism. Afterwards, participants were requested to indicate the health professionals involved in managing autism.

Participants had to choose which healthcare professionals would be involved in managing autism. Sixty-nine percent (*n* = 45) chose SLTs, followed by 17% (*n* = 11) who chose psychologists, and the remainder (*n* = 9) chose physicians, pharmacists or dentists. None of the participants chose multiple disciplines or indicated that treatment requires a multidisciplinary approach. Participants were asked to indicate how they felt about managing individuals with autism. The responses to the Likert-scale questions on the management of autism show that most (*n* = 53) of the 62 participants who responded to the question ‘*How do you feel about managing people with autism?*’ indicated that they were ‘neutral’, ‘positive’ or ‘very positive’ about managing individuals with autism. Only 14% (*n* = 9) of the 62 participants indicated either ‘I don’t know’ or felt ‘negative’ about managing individuals with autism. From the 24 participants who felt positive about managing individuals with autism, the majority (46%) (*n* = 11) were fourth-year students, which might be because most second- and third-year students have never been in contact with or had limited exposure to the disorder or received no information about autism. Only six (*n* = 6) second-year students and five (*n* = 5) third-year participants indicated they felt ‘very positive’ about managing individuals with autism.

Based on the quantitative findings concerning the management of autism, the participants presented adequate knowledge of the general aspects related to the treatment of autism. Participants also displayed limited knowledge concerning a multidisciplinary approach, including specific team members involved in managing autism. However, they displayed adequate knowledge that early intervention was specifically important in managing autism and that different treatment options should be available. Nevertheless, students displayed insufficient knowledge of the specific treatment options available, including augmentative and alternative communication (AAC), and behavioural interventions, for example, functional communication training (FCT) and discrete trial training (DTT). Limited knowledge concerning specific facilitation techniques, such as incidental teaching and milieu therapy, was noticed.

The results for the three open-ended questions related to the qualitative domain for Objective 3 are integrated and presented in Table 4. The response categories for these questions are summarised and presented. Some responses are not reflected in the response categories because of the participants’ inability to answer the specific questions; for example, 27% (*n* = 11) of the participants answered the questions with a ‘yes’ or ‘no’, although they had to provide a reason or opinion. The response categories to the open-ended questions related to the ‘importance of a team approach’, the ‘roles of SLTs in the management of autism’ and ‘participants’ feelings associated with the management of individuals with autism’ are summarised in Table 4. Some participants either answered the question inadequately or left the question unanswered.

From the 41 participants who answered the open-ended question ‘Why autism requires a team approach?’, 75.6% (*n* = 31) of the participants highlighted that autism requires a team approach to manage individuals. This response indicates that all functional areas, such as intellectual, neuropsychological, communicative, behavioural, adaptive behaviour and emotional functioning, should be managed through a multidisciplinary approach that will impact daily living skills and the quality of life of individuals with autism (Goldstein & Ozonoff 2018). According to Sinai-Gavrilov et al. (2019), autism impacts various developmental areas, requiring intervention and collaboration between professionals from multiple disciplines. In agreement, 9.76% (*n* = 4) also reported that collaboration between different team members is essential, and the remaining participants (4.8%) stated the importance of a team approach. Consequently, the most prominent theme was the ‘holistic management of autism’, which emphasises collaboration between different health professionals as they may recommend different but affirmative intervention approaches and strategies (Paynter et al. 2018).

Furthermore, most participants responded adequately to the open-ended question related to the ‘roles of SLTs in the management of autism’. Eighty-one percent (*n* = 31) reported that the role of SLTs is to improve language and communication difficulties in individuals with autism. Participants in the present study show an understanding that language and communication intervention forms part of the core responsibilities of SLTs in managing autism. However, only 16% of the participants reported that the SLTs’ role in the management of autism includes improvement of social interaction (i.e. behaviour, play and attention span). Consequently, participants’ most prominently mentioned theme regarding ‘*Development of communication and related aspects*’ is that SLTs assist with developing communication and social abilities.

Participants were encouraged to explain their feelings on managing individuals with autism. Even though 44 participants responded to this question, only 24 of the responses were considered. Sixteen (*n* = 16) or 66.7% of the participants felt inadequate and reported not having enough exposure and training to manage individuals with autism. Fifty percent (*n* = 8) of these respondents were from the fourth-year group, even though they have received training on autism. In contrast, only 33.3% (*n* = 8) of the participants who answered the question, primarily fourth-year students, felt positive about managing individuals with autism because they had gained enough experience. The responses to this question likewise do not correspond with the findings, as obtained from the Likert-type scale, indicating that participants were unsure of their competency in managing individuals with autism. The responses may indicate developing knowledge of the disorder, although it has not been established, specifically for ‘younger’ or more inexperienced students.

As seen in the findings earlier, responses obtained from the qualitative domain mostly validated the findings of the quantitative domain. After that, the results associated with Objective 4 reflect the SLPA students’ needs for additional training on autism.

The researchers explored whether the participants believed they received or were receiving enough training and if they needed more training on managing autism. For the quantitative results, a Likert-type scale and closed-ended questions were used to determine whether they ‘strongly disagree’, ‘disagree’, are ‘neutral’, ‘agree’ or ‘strongly agree’ with the above.

Twenty-eight (*n* = 28) or 43.8% of the 64 participants who responded to the question indicated that they were ‘neutral’ concerning this matter, whereas 19 or 29.5% indicated that they ‘disagree’ or ‘strongly disagree’ that students receive enough training on autism. The results show that the majority (*n* = 58, or 92%) of the participants indicated they would like additional training on autism. They agreed that additional training could improve their attitudes and perspectives on managing the disorder.

Based on the quantitative results obtained in Objective 4, almost an equal number of participants either ‘agree’ or ‘disagree’, and a high percentage were neutral that students at this Health Science University receive enough training on autism. Even though the results indicate that the students might be indecisive about whether they receive enough training, they indicated a need for additional education and training to improve students’ attitudes towards managing autism.

The response categories for an additional open-ended question, namely, ‘Reasons why it is important to receive additional training’ are presented in Table 5. Some participants either answered the question inadequately or left the question unanswered.

Only 37 participants answered the open-ended question. The results reflect that 62.2% (*n* = 23) highlighted that additional training on autism would enhance knowledge and improve competence in assessing and managing the disorder, and 8% (*n* = 14) of the participants indicated that additional training would increase their knowledge holistically. Hence, the most prominent theme from the identified categories was that training would lead to ‘enhanced proficiency’ in the holistic management of individuals with autism. Therefore, the theme validates the quantitative findings for Objective 4.

## Discussion

The demographics for the specific health sciences department might differ from similar departments from other universities as 19 students had a higher average age (24 – 28 years). The high percentage of older students may be because of a high failure rate; students may have studied another course, such as a bachelor’s degree in science, before registering for this specific health sciences course or may have entered university later because of financial constraints.

The results displayed adequate knowledge of the general aspects of autism and acceptable knowledge of the core symptoms (communication and behavioural difficulties) and the comorbidities of autism, such as ADHD. However, the students had limited knowledge of the prevalence and causes of autism, such as exposure to heavy metals and environmental toxins. Zerbo et al. (2015) and Chansa-Kabali et al. (2019) found similar results on healthcare professionals’ knowledge of autism.

Rohanachandra, Prathapan and Amarabandu (2020) reported that some health professionals in their study incorrectly believed that autism is caused by poor attention from parents and parental conflict. In the present study, students indicated that the impact of an individual’s environment in the early years does not contribute to the development of autism. However, some students in the study incorrectly believed that autism can be cured, and this corresponds with the research of Ellias and Shah (2019), who also identified knowledge gaps among healthcare students concerning the fact that autism is a lifelong condition.

Atun-Einy and Ben-Sasson (2018) additionally reported gaps among healthcare professionals on associated comorbidities, and Rohanachandra et al. (2017) emphasised the importance of sound knowledge and awareness among healthcare professionals relating to the features and comorbidities of autism. In early infancy, symptoms of autism are not always identified, which impacts early recognition and diagnoses during initial consultations (Matenge 2014). If SLTs are unaware of such complexities, they may fail to see the need for thorough and timely assessment. This negatively impacts early intervention and the quality of life of children with autism and their caregivers.

The findings of this study also show that students do not have adequate knowledge of diagnosing autism. However, participants knew that behaviour and general development be considered when diagnosing a child with autism. According to Anagnostou et al. (2014), the key to accurate diagnosis is a clear understanding of skills and deficits in social communication, behaviour and general development. Past studies in low- and lower-middle-income countries have also shown a shortfall of knowledge on the assessment of neurodevelopmental disorders such as autism among healthcare professionals (Ellias & Shah 2019), including the manner of identification and assessment, the interview structure and the use of diagnostic tools (Penner et al. 2018). Matenge (2014) and Atun-Einy and Ben-Sasson (2018) likewise indicated that healthcare professionals struggle to identify young children even though they should be at the forefront of the early identification of children at risk for autism. Hartley-McAndrew, Doody and Mertz (2016) mentioned a lack of knowledge of specific diagnostic criteria in the new DSM-5 and screening measures. Speech-language therapists’ incomplete knowledge of aspects related to the assessment of autism, in domains related to communication, language, speech and social behaviours, negatively impacts the identification of younger individuals and infants with autism (Matenge 2014; Vitaskova & Kytnarova 2017). Speech-language therapists also did not indicate that assessment and treatment require a multidisciplinary approach, which may also indicate a gap in knowledge relating to the complexity of the disorder (Yates & Le Couteur 2016). Health professionals’ lack of knowledge can additionally lead to misdiagnosis or late diagnosis that may result in poor outcomes and prognosis and will consequently impact the management of autism. Early diagnosis and monitoring of developmental milestones is important as it could result in early intervention that will improve the adaptive behaviour, functional communication and quality of life for autistic individuals and their caregivers (Bultas & Koetting 2014; Rohanachandra et al. 2017). Zwaigenbaum et al. (2015) further postulate that early identification and intervention minimise the progressive development of autism symptoms such as persistent social interaction challenges, speech and nonverbal communication problems, restricted, repetitive behaviours and auditory processing barriers.

The quantitative findings concerning the management of autism mostly correlate with the qualitative results. The qualitative results indicate that most students recognise the importance of a team approach even though they were not confident in utilising a multidisciplinary approach. They also did not adequately understand what the different roles of SLTs entail in managing communication and social abilities. However, a few students felt confident in managing individuals with autism because of some clinical exposure. The findings may indicate developing knowledge and management of the disorder, specifically for ‘younger’ students.

Findings of a study by Zerbo et al. (2015) also reported that various health and healthcare professionals rated their knowledge of providing treatment to individuals with autism as poor, although responses varied according to the type of health provider. The HCPs in Zerbo et al.’s (2015) research also reported that the lack of knowledge was because of receiving little or no training on managing the disorder during their years of undergraduate study, affecting the services they provide and leading to frustration (Martínez-Cayuelas et al. 2017). A study by Ghaderi and Watson (2019) emphasised that HCPs rate undergraduate training as useful, as it provides input and exposure to different autism-related cases. These researchers indicated that additional training would be beneficial to holistically improve HCPs’ knowledge and competence in providing care for individuals with autism, as many HCPs may have only received one lecture on autism during their undergraduate training. This negatively impacted their attitude and readiness to provide adequate services and suitable patient care to individuals with autism once they enter the formal work environment. These HCPs felt obliged to educate themselves on managing autism by attending conferences, workshops and training after graduating. They additionally indicated that extra lectures and training, including the identification, diagnoses, assessment and specifically the management of the disorder on an undergraduate level, would be beneficial.

This study recognised the need for students to receive further training on identifying, assessing, diagnosing and managing autism. The results correlate with the studies carried out by Havercamp et al. (2016) and Ghaderi and Watson (2019) identified a need for specific training for SLTs on identifying, diagnosing and treating autism because of the symptomology associated with autism. A global study conducted by Dillenburger et al. (2016) also documented the need for training HCPs as they had limited clinical exposure during their undergraduate training on the social stigma associated with this developmental disorder, effective communication and improved treatment outcomes for individuals with autism.

The outcome of an African study by Bakare et al. (2015) indicated that knowledge of the management of autism among final-year undergraduate healthcare students in Enugu, Nigeria, was poor. However, this study suggested that final-year undergraduate SLT students had the highest total mean score of all healthcare students. The discrepancy in knowledge found among the different cohorts of healthcare students was due to the variation in the hours of lectures presented to speech-language therapy, medical, and psychology students during their undergraduate training. Therefore, it is essential to implement suitable clinical training and residency programmes on autism as they will provide HCPs, such as SLTs, opportunities to enhance their competency and effective care delivery to autistic individuals (Ghaderi & Watson 2019; Martínez-Cayuelas et al. 2017). These findings support the need for student training on autism.

### Limitations and recommendations

The following limitations impacted the study negatively and have implications for future research, curriculum development and training of undergraduate students.

Obtaining information from the participants was challenging as the research was conducted online during the COVID-19 lockdown period, which negatively influenced the response rate. The researchers had to send reminders to the participants to encourage them to complete the questionnaire as most of them did not complete it timeously. Consequently, this might have impacted the reliability of the participants’ responses. Some open-ended questions were also not completed as the participants were required to provide their own opinions and reasons, possibly because it took more time to complete than closed-ended questions. The sample size varied for the different open-ended questions and reflected a small sample size. Hence, the results cannot be generalised.

In addition, because of the uneven distribution of students per year group, the results for the different groups were not compared. This was, however, not stated as an objective. In addition, more fourth-year students participated in the study. This could have influenced the results as fourth-year students may have more knowledge than the other year groups and have been providing services to autistic children.

In future, researchers should compare the knowledge between the different year groups or the different levels within the population to appreciate knowledge differences and determine the amount of knowledge obtained through formal teaching or as a result of personal study and experience.

## Conclusion

This study determined the current knowledge on autism among SLP & A students at a South African Higher Education Institution. The results of the study answered the research question, ‘What is the knowledge among students in the Department of SLP & A on autism?’ The participants have acceptable knowledge in certain areas pertaining to autism but limited knowledge in others. As indicated in the results and discussion sections, the quantitative and qualitative results suggest that the participants have adequate knowledge of core symptoms, general aspects related to autism and comorbidities. The results also suggest that the participants have limited knowledge of the prevalence and causes, diagnosis and the specific professionals responsible for the final diagnosis of autism. The quantitative and qualitative results of Objective 4 validate the results for the other objectives.

Furthermore, the participants had adequate knowledge of the importance of early intervention, the team members involved and the specific roles of SLTs in managing autism. They only partially understand specific treatment approaches and the overall management of autism. These outcomes may relate to the current curriculum, time constraints and insufficient clinical exposure. Hence, students reported the need for additional training on autism, specifically the identification, diagnosis, assessment and treatment of autism.
